# Relationship between Soft Drink Consumption and Obesity in 9–11 Years Old Children in a Multi-National Study

**DOI:** 10.3390/nu8120770

**Published:** 2016-11-30

**Authors:** Peter T. Katzmarzyk, Stephanie T. Broyles, Catherine M. Champagne, Jean-Philippe Chaput, Mikael Fogelholm, Gang Hu, Rebecca Kuriyan, Anura Kurpad, Estelle V. Lambert, Jose Maia, Victor Matsudo, Timothy Olds, Vincent Onywera, Olga L. Sarmiento, Martyn Standage, Mark S. Tremblay, Catrine Tudor-Locke, Pei Zhao

**Affiliations:** 1 Pennington Biomedical Research Center, Baton Rouge, LA 70808, USA; stephanie.broyles@pbrc.edu (S.T.B.); catherine.champagne@pbrc.edu (C.M.C.); gang.hu@pbrc.edu (G.H.); 2 Children’s Hospital of Eastern Ontario Research Institute, Ottawa, ON K1H 8L1, Canada; jpchaput@cheo.on.ca (J.-P.C.); mtremblay@cheo.on.ca (M.S.T.); 3 Department of Food and Environmental Sciences, University of Helsinki, Helsinki 00014, Finland; mikael.fogelholm@helsinki.fi; 4 St. Johns Research Institute, Bangalore 560034, India; rebecca@sjri.res.in (R.K.); a.kurpad@sjri.res.in (A.K.); 5 Division of Exercise Science and Sports Medicine, Faculty of Health Sciences, University of Cape Town, Newlands, Cape Town 7700, South Africa; vicki.lambert@uct.ac.za; 6 Faculdade de Desporto, University of Porto, Rua Dr. Plácido Costa, 91, Porto 4200-450, Portugal; jmaia@fade.up.pt; 7 Centro de Estudos do Laboratório de Aptidão Física de São Caetano do Sul, Sao Paulo 09520-320, Brazil; matsudo@celafiscs.org.br; 8 School of Health Sciences, Sansom Institute, University of South Australia, Adelaide, SA 5001, Australia; timothy.olds@unisa.edu.au; 9 Department of Recreation Management and Exercise Science, Kenyatta University, Nairobi 00100, Kenya; vonywera@gmail.com; 10 School of Medicine, Universidad de los Andes, Bogota 11001000, Colombia; osarmien@uniandes.edu.co; 11 Department for Health, University of Bath, Bath BA2 7AY, UK; m.standage@bath.ac.uk; 12 Department of Kinesiology, University of Massachusetts, Amherst, MA 01003, USA; ctudorlocke@umass.edu; 13 Tianjin Women’s and Children’s Health Center, Tianjin 300070, China; juliapeizhao@yeah.net

**Keywords:** pediatric, overweight, global, sugar-sweetened beverages

## Abstract

The purpose of this study was to determine the association between regular (sugar containing) and diet (artificially sweetened) soft drink consumption and obesity in children from 12 countries ranging in levels of economic and human development. The sample included 6162 children aged 9–11 years. Information on soft drink consumption was obtained using a food frequency questionnaire. Percentage body fat (%BF) was estimated by bio-electrical impedance analysis, body mass index (BMI) *z*-scores were computed using World Health Organization reference data, and obesity was defined as a BMI > +2 standard deviations (SD). Multi-level models were used to investigate trends in BMI *z*-scores, %BF and obesity across categories of soft drink consumption. Age, sex, study site, parental education and physical activity were included as covariates. There was a significant linear trend in BMI *z*-scores across categories of consumption of regular soft drinks in boys (*p* = 0.049), but not in girls; there were no significant trends in %BF or obesity observed in either boys or girls. There was no significant linear trend across categories of diet soft drink consumption in boys, but there was a graded, positive association in girls for BMI *z*-score (*p* = 0.0002) and %BF (*p* = 0.0001). Further research is required to explore these associations using longitudinal research designs.

## 1. Introduction

The worldwide prevalence of childhood overweight and obesity increased significantly between 1980 and 2013 in both developed and developing countries [1]. The obesity epidemic is hypothesized to have resulted from a complex web of international, national, local and intra-individual factors that have fostered long-term positive energy balance in an increasing proportion of the population [2]. At the individual level, energy balance is maintained largely by the interplay between energy intake and energy expenditure. A recent trend analysis of time-use data has documented global decreases in levels of physical activity [3]. However, long-term trends in dietary intake are difficult to assess, especially at the global level. Data from several countries corroborate the existence of a nutritional transition as countries have shifted towards a more “westernized” diet, albeit at different rates of progression [4]. A systematic assessment of trends in diet quality in 187 countries between 1990 and 2010 has concluded that the consumption of healthy food items has increased modestly in this time frame across the globe; however, the consumption of unhealthy food items increased to a greater extent [5]. Data on global trends in dietary energy intake per se are unfortunately unavailable.

Given the high caloric density of sugar-sweetened beverages, there has been considerable interest in understanding the association between sugar-sweetened beverage consumption and obesity. This topic has prompted lively academic debates [6,7] and spurred the development of government policies targeting reductions in sugar-sweetened beverage consumption in some jurisdictions [8]. Two recent reviews of published systematic reviews both concluded that the consumption of sugar-sweetened beverages is related to obesity [9,10]. The review by Bes-Restollo and colleagues [10], which included 24 systematic reviews (18 included both adults and children; one included adults only; five included children only), concluded that sugar-sweetened beverage consumption was significantly related to the risk of obesity. The review by Keller and Bucher Della Torre [9] focused exclusively on 14 systematic reviews in childhood, and concluded that the majority of the reviews found a direct association between sugar-sweetened beverage consumption and obesity; however, more recently, well-conducted meta-analyses showed discrepant results. In response to the available evidence, the American Heart Association recently recommended that children and adolescents limit their intake of sugar-sweetened beverages to one or fewer 8 oz beverages per week [11].

Artificially sweetened (diet) beverages have emerged as an alternative to sugar-sweetened beverages. To date, the relationship between obesity and diet beverages has not received the same level of attention as sugar-sweetened beverages. A recent systematic review identified five published cohort studies on the association between artificially sweetened beverage consumption and obesity in youth [12]. Overall, the association remains inconclusive, as one study found a positive association, one study found a negative association, and the remaining three studies found no association [12]. More research is required to better understand the association between artificially sweetened beverage consumption and obesity in children.

Given that the majority of the published studies on beverage consumption and obesity have been conducted in high-income North American and European countries, the aim of this study was to determine the cross-sectional association between consumption of regular (sugar containing) and diet (artificially sweetened) soft drinks and obesity in 9–11 years old children from countries ranging in levels of economic and human development. We hypothesized that there would be a positive relationship between consumption of regular soft drinks and obesity, but not with consumption of diet soft drinks.

## 2. Materials and Methods

The sample included 6162 children 9–11 years of age from sites in 12 countries spanning a range of economic and human development (Australia, Brazil, Canada, China, Colombia, Finland, India, Kenya, Portugal, South Africa, United Kingdom, United States). The countries were stratified into three groups based on World Bank classifications of economic development: Low and Lower-Middle Income (Kenya and India); Upper-Middle Income (Brazil, China, Colombia, South Africa); and High Income (Australia, Canada, Finland, Portugal, United Kingdom, United States) [13]. The rationale, design and methods of the International Study of Childhood Obesity, Lifestyle and the Environment (ISCOLE) have previously been published elsewhere [14]. Further, data on site-specific prevalence of obesity and global associations between obesity and socio-economic status have also been published [15,16].

The Pennington Biomedical Research Center Institutional Review Board as well as Institutional/Ethical Review Boards at each site approved the protocol. Written informed consent was obtained from parents/legal guardians, and child assent was also obtained as required before participation. Data were collected between September 2011 and December 2013.

### 2.1. Soft Drink Consumption

Information on soft drink consumption was obtained using a food frequency questionnaire (FFQ) adapted from the Health Behaviour in School-aged Children Survey [14,17]. The FFQ asked about the consumption of “Regular cola or soft drinks that contain sugar”, and “Diet cola or diet soft drinks”, with response categories including never, less than once per week, once per week, 2–4 days per week, 5–6 days per week, once a day every day, and more than once a day. A recent study reported a reliability correlation of 0.40 for diet soft drinks and 0.61 for regular soft drinks, among 321 participants who repeated this FFQ after an average of 4.9 weeks [18]. Given the difficulties in accurately assessing total energy intake in children, we did not attempt to quantify this or include it as a covariate.

### 2.2. Ascertainment of Adiposity and Obesity

Body mass and percentage body fat (% body fat) were measured with the Tanita SC-240 bio-electrical impedance scale (Arlington Heights, IL, USA), which has demonstrated acceptable validity for field studies [19]. Height was measured with a Seca 213 portable stadiometer (Hamburg, Germany) [14]. The average of two measurements was used for analysis (a third measurement was obtained if the first two measurements were greater than 0.5 cm, 0.5 kg, or 2.0% body fat apart for body height, body mass and % body fat, respectively, and the average of the two closest measurements was used in analyses). The body mass index (BMI; kg/m^2^) was calculated, and BMI *z*-scores were computed using age- and sex-specific reference data from the WHO [20]. Participants were classified as obese (BMI *z*-score > +2 standard deviations [SD]) or non-obese (BMI *z*-score ≤ +2 SD) [20].

### 2.3. Covariates

Age, sex, study site, highest level of parental education and physical activity were included as covariates in analytic statistical models. Age was computed from birth and observation dates and sex was recorded on a demographic and family history questionnaire. Highest level of parental education was coded into three categories based on the highest level of education attained by either parent (did not complete high school/completed high school or some college/completed bachelors or postgraduate degree).

Physical activity data were obtained following a 24-h protocol using waist-worn accelerometers (Actigraph GT3X+; Pensacola, FL, USA). Participants were asked to wear the accelerometer for at least 7 days (plus an initial familiarization day and the morning of the final day), including weekend days [21]. After removal of nocturnal sleep time from the data file using a published algorithm [22,23], awake non-wear time was defined as at least 20 consecutive minutes of zero activity counts [24], and moderate-to-vigorous physical activity (MVPA) was defined as all activity ≥574 counts per 15 s [25,26]. Only participants with at least 4 valid days (10+ h of awake wear time), including at least one weekend day, were included in the sample. Based on the average of the monitored days, participants were classified as physically active (≥60 min/day) or inactive (<60 min/day), according to the recommendations of the WHO [27].

### 2.4. Statistical Analysis

Given that the data in this study are nested within schools and study sites, we employed multi-level models for all analyses [28,29]. Multi-level linear mixed models (SAS version 9.4, PROC MIXED) were used to investigate trends in mean BMI *z*-scores across categories of regular and diet soft drink consumption (never, less than once per week, once per week, 2–4 days per week, 5–6 days per week, and once a day or more). Note that the two highest categories of soft drink consumption from the questionnaire were combined for all participants due to small sample sizes (i.e., zero) in these categories in some countries. Generalized linear mixed models (SAS version 9.4, PROC GLMMIX) were used to assess the association between soft drink consumption and obesity (0 = no; 1 = yes) in terms of odds ratios, where the “none” category was used as the reference. Study sites were considered to have fixed effects, and schools nested within study sites were viewed as having random effects. The denominator degrees of freedom for statistical tests pertaining to fixed effects were calculated using the Kenward and Roger approximation [30]. Similarly, multi-level linear mixed models (SAS version 9.4, PROC MIXED) were used to investigate trends in mean % body fat across categories of regular and diet soft drink consumption. Each model included sex, age, highest parental education, meeting physical activity guidelines (0 = no; 1 = yes), and study site as covariates. Preliminary models that included only age, sex and study site as covariates yielded essentially the same results as the fully adjusted models so only the results of the fully adjusted models are presented for BMI *z*-scores. Sex-by-soft drink, meeting physical activity guidelines-by-soft drink interactions and World Bank classification-by-soft drink interactions were included to determine if associations differed by sex, physical activity level or level of economic development. The level of significance was set at *p* < 0.05 for all analyses.

## 3. Results

Descriptive characteristics of the sample are presented in Table 1. The mean age of the sample was 10.4 (SD 0.6) years, and mean BMI *z*-scores were 0.53 (1.30) in boys and 0.39 (1.21) in girls. A total of 13.0% of boys and 16.9% of girls indicated that they “never” consumed regular soft drinks, whereas 46.7% and 49.3% of boys and girls, respectively, reported never consuming diet soft drinks.

**Figure 1:**
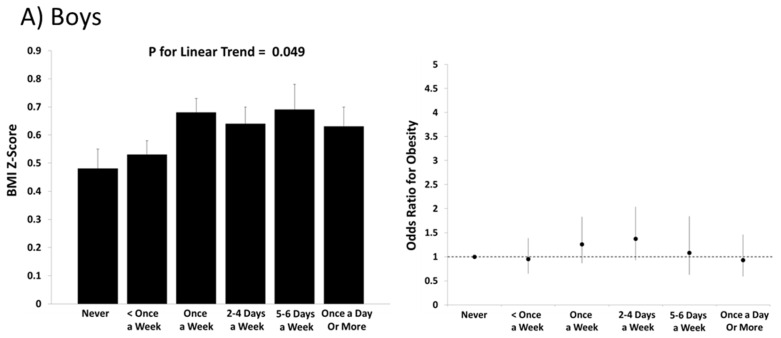

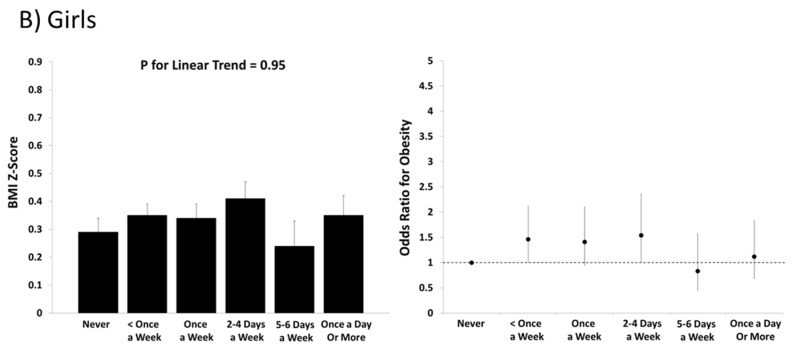
BMI *z*-scores and odds ratios for obesity across levels of consumption of regular soft drinks in (**A**) boys and (**B**) girls in the International Study of Childhood Obesity, Lifestyle and the Environment (ISCOLE). BMI *z*-scores were computed from World Health Organization reference data and associated error bars represent standard errors. Obesity was defined at >+2 standard deviations using World Health Organization reference data and associated error bars represent 95% confidence intervals. All models included age, study site, highest parental education and meeting physical activity guidelines as covariates.

**Figure 2:**
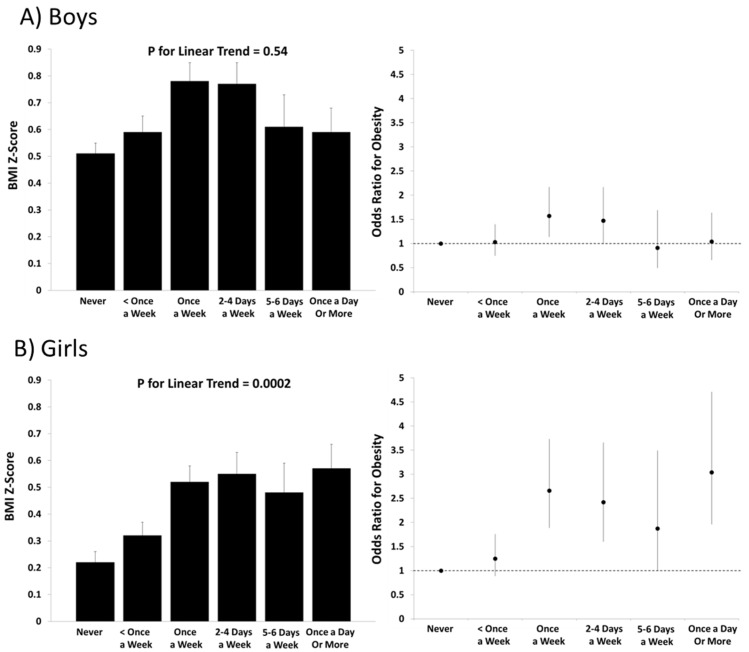
BMI *z*-scores and odds ratios for obesity across levels of diet soft drink consumption in (**A**) boys and (**B**) girls in the International Study of Childhood Obesity, Lifestyle and the Environment (ISCOLE). BMI *z*-scores were computed from World Health Organization reference data and associated error bars represent standard errors. Obesity was defined at >+2 standard deviations using World Health Organization reference data and associated error bars represent 95% confidence intervals. All models included age, study site, highest parental education and meeting physical activity guidelines as covariates.

In the total sample of boys and girls, there was no significant linear trend in BMI *z*-scores across levels of consumption of regular soft drinks (*p* = 0.27), but there was a significant positive trend across levels of consumption of diet soft drinks (*p* = 0.003). In all statistical models that included consumption of regular soft drinks as the outcome, the sex-by-soft drink interactions were not statistically significant (*p* = 0.38 to 0.63). When stratified by sex, there was a significant linear trend observed in boys (*p* = 0.049) but not in girls (*p* = 0.95) for consumption of regular soft drinks (Figure 1). For the diet soft drink consumption models, the sex-by-diet soft drink interactions were borderline or statistically significant (*p* = 0.13 to 0.001). There was no significant linear trend across levels of diet soft drink consumption in boys (*p* = 0.55) but there was a positive linear association in girls (*p* = 0.0002; Figure 2).

The results from the generalized mixed models for predicting obesity follow a similar pattern as the results for BMI *z*-scores. In boys, none of the categories of consumption of regular soft drinks had an elevated odds of obesity relative to the “never” category (Figure 1). In girls, there was an elevated odds of obesity (OR = 1.46 (95% CI: 1.00–2.13)) among those who reported drinking regular soft drinks less than once a week compared to the “never” category. In boys there was an elevated odds of obesity (1.57 (1.14–2.17)) among those who reported drinking diet soft drinks once a week compared to the “never” category (Figure 2). Among girls, several categories of diet soft drink consumption demonstrated a higher odds of obesity relative to the “never” category (once a week: 2.66 (1.89–3.73); 2–4 days a week: 2.42 (1.60–3.66); 5–6 days a week: 1.87 (1.01–3.49); and once a day or more: 3.04 (1.96–4.71)) (Figure 2).

Table 2 presents the results of the multi-level mixed models that tested the association between soft drink consumption and % body fat. There was no association between consumption of regular soft drinks and % body fat in boys (*p* = 0.13) or girls (*p* = 0.49); however, there was a positive association between diet soft drink consumption and % body fat in girls (*p* = 0.0001) but not in boys (*p* = 0.74). Note that the results are similar between the models that include highest parental education and physical activity as covariates versus those that do not.

The World Bank classification-by-consumption of regular soft drinks interaction was significant in boys (*p* = 0.02) but not in girls (*p* = 0.64) for BMI *z*-scores. Table 3 presents the results for BMI *z*-scores stratified by country-level income. In boys, there was a positive association between consumption of regular soft drinks and BMI *z*-scores in the low and lower-middle countries (*p* = 0.0001), but there was no associations in the other groups. On the other hand, the World Bank classification-by-diet soft drink consumption interaction was significant in girls (*p* = 0.01) but not in boys (*p* = 0.08). There were no associations between diet soft drink consumption in boys in any of the income groups; however, there was a positive association between diet soft drink consumption and BMI *z*-scores in girls from upper-middle (*p* = 0.01) and high income (*p* = 0.03) countries.

Supplementary Materials Table S1 presents the results of country-level analyses for BMI *z*-scores across levels of soft drink consumption. There was a significant positive trend across levels of consumption of regular soft drinks in boys in India (*p* < 0.0001) and a significant negative trend across levels of consumption of regular soft drinks in boys in South Africa (*p* = 0.01). In girls there were significant positive trend across levels of diet soft drink consumption in Canada (*p* = 0.02), Colombia (*p* = 0.03) and the United Kingdom (*p* = 0.04).

None of the physical activity-by-soft drink consumption interactions were significant in any model (*p* = 0.30 to 0.99) with the exception of consumption of regular soft drinks-by-physical activity in the GLMMIX model predicting obesity (*p* = 0.04).

## 4. Discussion

The consumption of sugar-sweetened beverages is common among children. The results from the present study indicate that 12.8% of boys and 10.8% of girls (Table 1) reported daily consumption of regular soft drinks. These results are similar to those reported for the 2013/2014 Health Behaviour in School Aged-Children Survey: 17% and 13% of 11-year old boys and girls, respectively, from 43 mainly European countries reported daily consumption of soft drinks [31]. Data from the United States indicate that sugar-sweetened beverage consumption increased in children between the 1988–1994 and 1999–2004 National Health and Nutrition Examination Surveys (NHANES) [32]; however, a more recent analysis indicates that energy intake from sugar-sweetened beverages in children declined approximately 30% between 1999 and 2010, from 223 kcal/day to 155 kcal/day [33].

In the present study, it was more common for children to report never drinking diet soft drinks than never drinking regular soft drinks. These results are consistent with data from the United States, where 6–11 years old children were more likely to report consuming at least 1 sugar-sweetened beverage (80%) compared to at least 1 diet beverage (5%) in the 1999–2004 NHANES [32].

Contrary to our hypothesis, this study did not find a robust association between consumption of regular soft drinks and obesity. There was a positive association observed in boys (*p* = 0.049); however, this result was driven by a strong association in Indian boys but not at other sites (see Supplementary Materials Table S1). The results from other population-based studies in children have shown mixed results with respect to the relationship between sugar-sweetened beverages and obesity. Our results are similar to those reported from an analysis of data from 204,534 children aged 11–15 years from 41 countries participating in the Health Behaviour in School-Aged Children Survey, in which soft drink consumption was not associated with being overweight [34]. Further, there was no association between sugar-sweetened beverage consumption and changes in BMI and waist circumference between the ages of 9 and 21 years in the Danish cohort of the European Youth Heart Study; however, at age 15 years, those who consumed more than one serving of sugar-sweetened beverages had greater increases in BMI and waist circumference by the age of 21 [35]. On the other hand, analyses from the 1999 to 2004 NHANES indicated a positive association between sugar-sweetened beverage consumption and BMI percentile in 12–19 years old boys and girls [36].

A positive association was observed between diet soft drink consumption and obesity in girls. This was an unexpected finding given that diet soft drinks do not contain any calories. However, previous studies have also reported a positive association between diet beverage consumption and weight gain in adolescents [37,38]. There are two potential explanations for this observation. First, it has been suggested that exposure to artificial sweeteners may increase appetite sensations and food intake in some cases [39]; however, most studies have failed to show an association between artificial sweeteners and food intake [40]. Second, given that this study is cross-sectional, reverse causation may be playing a role; it may be that obese girls are preferentially selecting diet soft drinks in an attempt to control their weight. In general, the relationship between diet beverage consumption and obesity in children is not well understood, and more research is required using longitudinal and intervention designs.

This study included sites from countries varying widely in levels of economic and human development. Significant interactions were observed between level of economic development and soft drink consumption. Stratified analyses revealed that the positive association between consumption of regular soft drinks and BMI *z*-scores in boys was limited to the group of low and low-middle income countries (Kenya and India), and further stratification by study site revealed that the positive association was only significant in boys from India. On the other hand, the strong positive association observed between diet soft drink consumption and BMI *z*-score (and % body fat and obesity) in girls was predominantly observed in upper-middle and high income countries. Although the site-level sample sizes in individual sites of ISCOLE may not be sufficient to identify significant associations, these results suggest that further research on the association between soft drink consumption and obesity should be performed in different contexts such as those found in developing countries, especially since the majority of the existing research has been conducted in high income countries.

This study has several strengths and limitations that warrant discussion. Two major strengths include the large sample of children from a diverse group of countries, and the standardized measurement of BMI and % body fat across all study sites. However, a limitation is the relatively crude FFQ used to assess beverage consumption. The validity of the FFQ used in ISCOLE has been studied [18]. Based on correlations between the FFQ and a pre-coded food diary, and on gross misclassifications of the consumption frequency, the FFQ used in ISCOLE compares well with other FFQ’s used to assess food consumption in children. Yet, the validity of consumption frequency of many of the food groups was only poor or moderate, and this error may increase the likelihood of type II error. The correlations between consumption frequency of soft drinks and other food groups were weak in this sample. Nevertheless, in a principal components analysis used to study dietary patterns among the ISCOLE participants, both regular and diet soft drinks loaded in the “unhealthy dietary pattern” factor [41]. Hence, together with the inaccuracy of the frequency assessment [18], there might be some residual confounding remaining from the collinearity between unhealthy food items. We relied on the WHO growth reference data to compute BMI *z*-scores and define obesity in the sample. These reference data are based on the original US National Center for Health Statistics data [20], and the degree to which this introduces bias when used in other countries is not known. A further limitation of this study is the cross-sectional research design, which precludes any cause-and-effect interpretation of the observed associations, and increases the potential for reverse causation as an explanation for the observations. Furthermore, we cannot exclude the possibility that unmeasured confounding variables may explain some of the observed relationships. We were unable to adjust for potential confounders such as biological maturity, total dietary intake, and other potential obesity-related factors.

## 5. Conclusions

In conclusion, the results of this large multinational study of children found a weak positive association between the consumption of regular soft drinks and obesity in boys, which was largely limited to boys from India. A significant positive association was observed between diet soft drink consumption and obesity in girls, but the cross-sectional design of the study precludes a definitive interpretation of this relationship. Further studies using longitudinal research designs are needed to better understand the prospective associations among beverage consumption and obesity in children.

## Figures and Tables

**Table 1:** Descriptive statistics of participants involved in the International Study of Childhood Obesity, Lifestyle and the Environment (ISCOLE).

	Boys	Girls
*N*	2815	3347
Age (year)	10.4 (0.6)	10.4 (0.6)
BMI (kg/m^2^)	18.4 (3.4)	18.4 (3.5)
BMI *z*-score	0.53 (1.30)	0.39 (1.21)
Body Fat (%)	18.9 (7.2)	22.5 (7.7)
Physical Activity (min/day)	69.8 (25.8)	52.3 (20.9)
Meeting Physical Activity Guidelines (%)	61.1	30.4
Obesity (%) *	15.1	10.1
Consumption of Regular Soft Drinks (%)		
Never	13.0	16.9
Less than once a week	24.4	29.7
Once a week	24.3	22.2
2–4 days a week	18.8	14.8
5–6 days a week	6.6	5.7
Once a day or more	12.8	10.8
Consumption of Diet Soft Drinks (%)		
Never	46.7	49.3
Less than once a week	19.8	21.1
Once a week	14.0	12.9
2–4 days a week	8.6	7.6
5–6 days a week	3.8	3.3
Once a day or more	7.1	5.9
Highest Parental Education (%)		
Did not complete high school	19.3	20.1
Completed high school or some college	43.1	41.8
Completed bachelors or postgraduate degree	37.7	38.2

BMI: Body mass index; * Obesity was defined as a BMI *z*-score > +2 standard deviations from the World Health Organization reference data [20].

**Table 2:** Results of multi-level mixed models * testing differences in % body fat (mean ± S.E.) across levels of soft drink consumption in the International Study of Childhood Obesity, Lifestyle and the Environment (ISCOLE).

	Boys	Girls
Regular Soft Drink Consumption		
Never	18.8 ± 0.4	22.0 ± 0.4
Less than once a week	19.0 ± 0.3	22.2 ± 0.3
Once a week	19.7 ± 0.3	22.2 ± 0.3
2–4 days a week	19.6 ± 0.3	22.5 ± 0.4
5–6 days a week	19.4 ± 0.5	21.4 ± 0.5
Once a day or more	19.5 ± 0.4	21.9 ± 0.4
*p* for Trend	0.13	0.49
Diet Soft Drink Consumption		
Never	18.9 ± 0.2	21.4 ± 0.3
Less than once a week	19.5 ± 0.3	21.8 ± 0.3
Once a week	19.9 ± 0.4	23.2 ± 0.4
2–4 days a week	20.3 ± 0.5	23.5 ± 0.5
5–6 days a week	19.0 ± 0.7	23.1 ± 0.7
Once a day or more	19.3 ± 0.5	23.5 ± 0.5
*p* for Trend	0.74	0.0001

* Means are adjusted for age, sex, study site, highest level parental education and meeting moderate-to-vigorous physical activity guidelines; S.E. = standard error.

**Table 3:** Results of multi-level mixed models testing for linear trends in BMI *z*-scores (mean ± S.E.) across levels of soft drink consumption in boys and girls in the International Study of Childhood Obesity, Lifestyle and the Environment (ISCOLE) stratified by country-level World Bank classification of economic status.

	*N*	None	<Once/Week	Once/Week	2–4 Days/Week	5–6 Days/Week	≥Once a Day	*p* *
	*Consumption of Regular Soft Drinks*							
Boys								
Income Category								
Low & Lower-Middle	474	−0.32 ± 0.16	0.06 ± 0.14	0.07 ± 0.14	0.31 ± 0.20	0.90 ± 0.27	0.28 ± 0.22	0.0001
Upper-Middle	1051	0.73 ± 0.13	0.68 ± 0.10	0.89 ± 0.09	0.67 ± 0.09	0.68 ± 0.15	0.66 ± 0.10	0.55
High	1290	0.65 ± 0.10	0.59 ± 0.07	0.71 ± 0.07	0.73 ± 0.08	0.62 ± 0.13	0.76 ± 0.11	0.47
Girls								
Income Category								
Low & Lower-Middle	554	−0.19 ± 0.13	−0.20 ± 0.11	−0.13 ± 0.12	−0.10 ± 0.20	−0.25 ± 0.22	0.11 ± 0.19	0.29
Upper-Middle	1132	0.40 ± 0.10	0.41 ± 0.08	0.29 ± 0.08	0.31 ± 0.09	0.21 ± 0.14	0.25 ± 0.10	0.11
High	1661	0.40 ± 0.07	0.50 ± 0.05	0.52 ± 0.06	0.62 ± 0.08	0.42 ± 0.13	0.48 ± 0.11	0.71
	*Consumption of Diet Soft Drinks*							
Boys								
Income Category								
Low & Lower-Middle	474	−0.05 ± 0.13	0.41 ± 0.18	0.14 ± 0.17	0.23 ± 0.25	−0.16 ± 0.35	0.07 ± 0.21	0.52
Upper-Middle	1051	0.68 ± 0.06	0.72 ± 0.11	1.00 ± 0.12	0.74 ± 0.16	0.73 ± 0.22	0.58 ± 0.15	0.53
High	1290	0.58 ± 0.06	0.58 ± 0.07	0.82 ± 0.09	0.91 ± 0.10	0.72 ± 0.16	0.78 ± 0.13	0.09
Girls								
Income Category								
Low & Lower-Middle	554	−0.33 ± 0.10	−0.13 ± 0.15	0.17 ± 0.16	−0.17 ± 0.23	−0.05 ± 0.24	0.15 ± 0.18	0.09
Upper-Middle	1132	0.30 ± 0.06	0.23 ± 0.10	0.29 ± 0.11	0.27 ± 0.14	0.60 ± 0.20	0.63 ± 0.15	0.01
High	1661	0.31 ± 0.05	0.48 ± 0.06	0.76 ± 0.08	0.89 ± 0.10	0.56 ± 0.17	0.61 ± 0.13	0.03

* *p* for linear trend test across categories of soft drink consumption. Means are adjusted for age, sex, study site, highest level parental education and meeting moderate-to-vigorous physical activity guidelines.

## Supplementary Materials

The following are available online at http://www.mdpi.com/2072-6643/8/12/770/s1, Table S1: Results of multi-level mixed models testing for linear trends in BMI *z*-scores (mean ± S.E.) across levels of soft drink consumption in boys and girls in the International Study of Childhood Obesity, Lifestyle and the Environment (ISCOLE) stratified by study site.
