# Supplementary material for: Relationship between Soft Drink Consumption and Obesity in 9–11 Years Old Children in a Multi-National Study
Source: Nutrients. 2016 Nov 30;8(12):770. doi: 10.3390/nu8120770 (PMC5188425; doi:10.3390/nu8120770)
Supplement: Supplementary file 1 [file nutrients-08-00770-s001.docx]

**Supplementary Materials: Relationship between Soft Drink Consumption and Obesity in 9–11 Years Old Children in a Multi-National Study**

Peter T. Katzmarzyk, Stephanie T. Broyles, Catherine M. Champagne, Jean-Philippe Chaput, Mikael Fogelholm, Gang Hu, Rebecca Kuriyan, Anura Kurpad, Estelle V. Lambert, Jose Maia, Victor Matsudo, Timothy Olds, Vincent Onywera, Olga L. Sarmiento, Martyn Standage,
Mark S. Tremblay, Catrine Tudor-Locke and Pei Zhao

**Table S1.** Results of multi-level mixed models testing for linear trends in BMI z-scores (mean ± S.E.) across levels of soft drink consumption in boys and girls in the International Study of Childhood Obesity, Lifestyle and the Environment (ISCOLE) stratified by study site.

|  | ***N*** | **None** | **<Once/Week** | **Once/Week** | **2–4 Days/Week** | **5–6 Days/Week** | **≥Once a Day** | ***p* *** |
| --- | --- | --- | --- | --- | --- | --- | --- | --- |
|  | *Consumption of Regular Soft Drinks* | | | | | | | |
| Boys |  |  |  |  |  |  |  |  |
| Australia | 220 | 0.84 ± 0.25 | 0.54 ± 0.15 | 0.98 ± 0.17 | 0.86 ± 0.19 | 0.76 ± 0.30 | 1.33 ± 0.32 | 0.17 |
| Brazil | 216 | 1.06 ± 0.41 | 1.36 ± 0.27 | 1.51 ± 0.24 | 0.90 ± 0.24 | 0.67 ± 0.37 | 1.00 ± 0.22 | 0.25 |
| Canada | 206 | 0.84 ± 0.26 | 0.77 ± 0.23 | 0.82 ± 0.29 | 1.12 ± 0.25 | 1.67 ± 0.46 | −0.13 ± 0.50 | 0.54 |
| China | 258 | 0.74 ± 0.23 | 0.77 ± 0.21 | 1.18 ± 0.20 | 0.97 ± 0.30 | 1.18 ± 0.54 | 1.12 ± 0.33 | 0.27 |
| Colombia | 422 | 0.61 ± 0.17 | 0.33 ± 0.13 | 0.57 ± 0.12 | 0.42 ± 0.11 | 0.61 ± 0.18 | 0.46 ± 0.14 | 0.97 |
| Finland | 217 | 0.64 ± 0.28 | 0.27 ± 0.22 | 0.52 ± 0.21 | 0.44 ± 0.24 | 0.14 ± 0.40 | 0.93 ±0.77 | 0.83 |
| India | 249 | −0.79 ± 0.22 | −0.10 ± 0.18 | −0.31 ± 0.19 | 0.07 ± 0.30 | 1.13 ± 0.36 | 0.19 ± 0.32 | <0.0001 |
| Kenya | 225 | 0.03 ± 0.22 | 0.01 ± 0.19 | 0.27 ± 0.17 | 0.36 ± 0.24 | 0.16 ± 0.38 | 0.23 ± 0.27 | 0.43 |
| Portugal | 269 | 0.75 ± 0.21 | 1.16 ± 0.18 | 0.99 ± 0.14 | 1.06 ± 0.17 | 0.57 ± 0.27 | 0.93 ± 0.20 | 0.66 |
| South Africa | 155 | 1.41 ± 0.44 | 0.65 ± 0.29 | 0.29 ± 0.25 | 0.53 ± 0.24 | 0.26 ± 0.32 | 0.27 ± 0.19 | 0.01 |
| United Kingdom | 187 | 0.61 ± 0.30 | 1.06 ± 0.22 | 1.03 ± 0.22 | 1.04 ± 0.20 | 0.91 ± 0.27 | 0.99 ± 0.25 | 0.47 |
| United States | 191 | 1.00 ± 0.32 | 0.71 ± 0.24 | 0.73 ± 0.23 | 0.49 ± 0.24 | 0.83 ± 0.36 | 0.89 ± 0.24 | 0.85 |
| Girls |  |  |  |  |  |  |  |  |
| Australia | 257 | 0.54 ± 0.19 | 0.52 ± 0.13 | 0.37 ± 0.16 | 0.79 ± 0.17 | 0.28 ± 0.33 | 0.77 ± 0.24 | 0.66 |
| Brazil | 224 | 0.85 ± 0.30 | 1.07 ± 0.22 | 0.87 ± 0.22 | 0.80 ± 0.20 | 0.84 ± 0.33 | 0.33 ± 0.22 | 0.11 |
| Canada | 290 | 0.11 ± 0.23 | 0.12 ± 0.20 | 0.00 ± 0.25 | 0.54 ± 0.27 | 0.69 ± 0.39 | 1.17 ± 0.86 | 0.09 |
| China | 238 | 0.29 ± 0.22 | 0.37 ± 0.21 | −0.18 ± 0.23 | 0.41 ± 0.29 | 0.24 ± 0.49 | 0.78 ± 0.43 | 0.33 |
| Colombia | 434 | 0.12 ± 0.12 | 0.03 ± 0.11 | 0.11 ± 0.10 | 0.14 ± 0.11 | −0.23 ± 0.17 | 0.07 ± 0.12 | 0.33 |
| Finland | 248 | −0.04 ± 0.22 | 0.01 ± 0.16 | 0.12 ± 0.18 | 0.09 ± 0.21 | −0.09 ± 0.47 | −0.84 ± 0.61 | 0.20 |
| India | 297 | 0.03 ± 0.22 | 0.14 ± 0.23 | 0.29 ± 0.28 | −0.11 ± 0.37 | −0.16 ± 0.43 | 0.72 ± 0.45 | 0.38 |
| Kenya | 257 | 0.00 ± 0.22 | −0.29 ± 0.16 | −0.19 ± 0.14 | 0.01 ± 0.23 | −0.09 ± 0.26 | 0.00 ± 0.19 | 0.63 |
| Portugal | 350 | 0.53 ± 0.16 | 0.79 ± 0.13 | 0.84 ± 0.13 | 0.57 ± 0.16 | 0.57 ± 0.25 | 0.76 ± 0.22 | 0.88 |
| South Africa | 236 | 0.54 ± 0.37 | 0.32 ± 0.22 | 0.48 ± 0.20 | 0.09 ± 0.23 | 0.37 ± 0.29 | 0.38 ± 0.19 | 0.66 |
| United Kingdom | 243 | 0.19 ± 0.25 | 0.34 ± 0.19 | 0.42 ± 0.20 | 0.98 ± 0.21 | 0.53 ± 0.32 | 0.44 ± 0.26 | 0.20 |
| United States | 273 | 0.80 ± 0.22 | 1.00 ± 0.19 | 0.99 ± 0.21 | 0.74 ± 0.23 | 0.34 ± 0.32 | 0.64 ± 0.21 | 0.08 |
|  | *Consumption of Diet Soft Drinks* | | | | | | | |
| Boys |  |  |  |  |  |  |  |  |
| Australia | 220 | 0.65 ± 0.14 | 0.69 ± 0.15 | 1.13 ± 0.21 | 1.10 ± 0.24 | 0.69 ± 0.42 | 0.74 ± 0.40 | 0.85 |
| Brazil | 216 | 1.08 ± 0.18 | 1.08 ± 0.25 | 1.42 ± 0.31 | 1.15 ± 0.43 | 1.21 ± 0.59 | 1.03 ± 0.34 | 0.95 |
| Canada | 206 | 0.71 ± 0.22 | 0.61 ± 0.25 | 0.88 ± 0.37 | 1.03 ± 0.42 | 0.77 ± 0.68 | 1.64 ± 0.57 | 0.16 |
| China | 258 | 0.94 ± 0.17 | 0.79 ± 0.23 | 1.31 ± 0.27 | 0.98 ± 0.32 | 0.88 ± 0.61 | 0.61 ± 0.45 | 0.58 |
| Colombia | 422 | 0.44 ± 0.07 | 0.73 ± 0.23 | 0.71 ± 0.20 | 0.59 ± 0.28 | 0.31 ± 0.31 | 0.64 ± 0.29 | 0.83 |
| Finland | 217 | 0.28 ± 0.22 | 0.22 ± 0.20 | 0.79 ± 0.24 | 0.79 ± 0.26 | 0.44 ± 0.38 | 1.52 ± 0.77 | 0.09 |
| India | 249 | −0.24 ± 0.14 | 0.30 ± 0.28 | −0.28 ± 0.28 | 0.83 ± 0.64 | −0.50 ± 0.64 | 0.02 ± 0.46 | 0.99 |
| Kenya | 225 | 0.01 ± 0.18 | 0.41 ± 0.21 | 0.38 ± 0.19 | 0.10 ± 0.25 | 0.01 ± 0.37 | 0.05 ± 0.21 | 0.47 |
| Portugal | 269 | 0.84 ± 0.13 | 1.17 ± 0.18 | 0.98 ± 0.18 | 1.02 ± 0.19 | 0.83 ± 0.27 | 1.08 ± 0.24 | 0.88 |
| South Africa | 155 | 0.47 ± 0.20 | 0.49 ± 0.26 | 0.59 ± 0.26 | 0.27 ± 0.31 | 0.64 ± 0.37 | 0.12 ± 0.26 | 0.43 |
| United Kingdom | 187 | 0.87 ± 0.19 | 0.99 ± 0.21 | 0.79 ± 0.22 | 1.25 ± 0.24 | 1.22 ± 0.38 | 1.03 ± 0.32 | 0.36 |
| United States | 191 | 0.70 ± 0.19 | 0.53 ± 0.26 | 0.97 ± 0.30 | 0.89 ± 0.34 | 1.16 ± 0.46 | 0.69 ± 0.28 | 0.43 |
| Girls |  |  |  |  |  |  |  |  |
| Australia | 257 | 0.44 ± 0.13 | 0.57 ± 0.13 | 0.46 ± 0.18 | 1.07 ± 0.23 | 0.33 ± 0.33 | 1.12 ± 0.42 | 0.18 |
| Brazil | 224 | 0.76 ± 0.16 | 0.68 ± 0.24 | 0.84 ± 0.27 | 0.73 ± 0.30 | 0.65 ± 0.60 | 1.32 ± 0.45 | 0.40 |
| Canada | 290 | 0.15 ± 0.20 | 0.14 ± 0.21 | 0.57 ± 0.30 | 0.82 ± 0.38 | 1.79 ± 0.85 | 1.19 ± 0.71 | 0.02 |
| China | 238 | 0.26 ± 0.19 | −0.13 ± 0.23 | 0.40 ± 0.25 | 0.03 ± 0.38 | 0.76 ± 0.44 | 0.82 ± 0.45 | 0.06 |
| Colombia | 434 | 0.05 ± 0.06 | 0.05 ± 0.20 | 0.04 ± 0.21 | 0.01 ± 0.21 | 0.53 ± 0.69 | 1.00 ± 0.33 | 0.03 |
| Finland | 248 | −0.23 ± 0.17 | −0.02 ± 0.16 | 0.30 ± 0.21 | 0.42 ± 0.25 | −0.32 ± 0.72 | ---- | 0.86 |
| India | 297 | 0.03 ± 0.20 | 0.41 ± 0.30 | 0.61 ± 0.38 | 0.50 ± 0.47 | 0.29 ± 0.67 | 0.75 ± 0.61 | 0.38 |
| Kenya | 257 | −0.28 ± 0.15 | −0.33 ± 0.17 | 0.07 ± 0.17 | −0.32 ± 0.26 | −0.02 ± 0.24 | 0.08 ± 0.17 | 0.09 |
| Portugal | 350 | 0.60 ± 0.11 | 0.78 ± 0.15 | 0.90 ± 0.15 | 0.97 ± 0.20 | 0.82 ± 0.28 | 0.33 ± 0.21 | 0.44 |
| South Africa | 236 | 0.31 ± 0.17 | 0.60 ± 0.23 | 0.13 ± 0.21 | 0.41 ± 0.30 | 0.56 ± 0.30 | 0.34 ± 0.22 | 0.85 |
| United Kingdom | 243 | 0.07 ± 0.19 | 0.32 ± 0.18 | 0.84 ± 0.21 | 0.85 ± 0.22 | 0.55 ± 0.47 | 0.91 ± 0.35 | 0.04 |
| United States | 273 | 0.52 ± 0.16 | 1.05 ± 0.20 | 1.25 ± 0.25 | 1.11 ± 0.32 | 0.92 ± 0.45 | 1.05 ± 0.25 | 0.27 |

* *p* for linear trend test across categories of soft drink consumption. Means are adjusted for age, highest level parental education and meeting moderate-to-vigorous physical activity guidelines.
